# Morusin Suppresses Cancer Cell Invasion and MMP-2 Expression through ERK Signaling in Human Nasopharyngeal Carcinoma

**DOI:** 10.3390/molecules25204851

**Published:** 2020-10-21

**Authors:** Cheng-Chen Huang, Po-Hui Wang, Yen-Ting Lu, Jia-Sin Yang, Shun-Fa Yang, Yu-Ting Ho, Chiao-Wen Lin, Chung-Han Hsin

**Affiliations:** 1 Institute of Medicine, Chung Shan Medical University, Taichung 402, Taiwan; ph7222@hotmail.com (C.-C.H.); wang082160@yahoo.com.tw (P.-H.W.); ytlu.tw@gmail.com (Y.-T.L.); gazn_sheep@yahoo.com.tw (J.-S.Y.); ysf@csmu.edu.tw (S.-F.Y.); Tina20817@hotmail.com (Y.-T.H.); 2 Department of Otolaryngology, Chung Shan Medical University Hospital, Taichung 402, Taiwan; 3 Department of Obstetrics and Gynecology, Chung Shan Medical University Hospital, Taichung 402, Taiwan; 4 Department of Otolaryngology, St. Martin De Porres Hospital, Chiayi 600, Taiwan; 5 Department of Medical Research, Chung Shan Medical University Hospital, Taichung 402, Taiwan; 6 Institute of Oral Sciences, Chung Shan Medical University, Taichung 402, Taiwan; 7 Department of Dentistry, Chung Shan Medical University Hospital, Taichung 402, Taiwan; 8 School of Medicine, Chung Shan Medical University, Taichung 402, Taiwan

**Keywords:** morusin, nasopharyngeal carcinoma, metastasis, MMP-2

## Abstract

The most important cause of treatment failure of nasopharyngeal carcinoma (NPC) patients is metastasis, including regional lymph nodes or distant metastasis, resulting in a poor prognosis and challenges for treatment. In the present study, we investigated the in vitro anti- tumoral properties of morusin on human nasopharyngeal carcinoma HONE-1, NPC-39, and NPC-BM cells. Our study revealed that morusin suppressed the migration and invasion abilities of the three NPC cells. Gelatin zymography assay and Western blotting demonstrated that the enzyme activity and the level of matrix metalloproteinases-2 (MMP-2) protein were downregulated by the treatment of morusin. Mitogen-activated protein kinase proteins were examined to identify the signaling pathway, which showed that phosphorylation of ERK1/2 was inhibited after the treatment of morusin. In summary, our data showed that morusin inhibited the migration and invasion of NPC cells by suppressing the expression of MMP-2 by downregulating the ERK1/2 signaling pathway, suggesting that morusin may be a potential candidate for chemoprevention or adjuvant therapy of NPC.

## 1. Introduction

Flavonoids have demonstrated anti-tumor activity in many types of human cancer cells. Compounds such as chalcone, a flavonoid precursor, induced apoptosis in gastric cancer by upregulation of DR4 and DR5 expression [1]. Baicalein, another kind of flavonoids, has pharmacological anti-cancer ability in breast cancer [2]. It also inhibited the expression of vascular endothelial growth factor (VEGF) in human ovarian cancer [3]. Morusin is another kind of prenylated flavonoids purified from a medical plant. It was extracted from the root bark of *Morus australis* (*Moraceae*) and has anti-microbial ability as well as anti-inflammatory activity [4]. Furthermore, previous studies revealed that morusin possesses anti-cancer biological effect. For instance, morusin inhibits tumor growth in human gastric cancer in vitro and in vivo through downregulating c-Myc expression [5]. In human breast cancer, morusin induced apoptosis by decreasing Survivin and increasing Bax protein expression [6] and induces apoptosis through inhibiting signal transducer and activator of transcription 3 (STAT3) in the prostate cancer [7]. Besides, morusin also induced apoptosis in colorectal cancer by suppressing nuclear factor (NF)-κB signaling pathway [8].

Nasopharyngeal carcinoma (NPC) is endemic in some countries, especially in Southern China and Southeast Asia, with the incidence rate of 50 in 100,000 people [9]. Besides, a previous study demonstrated that the mortality of NPC in South China is still higher than any country in the world [10]. The primary curative therapy for nasopharyngeal carcinoma is radiotherapy. Intensity-modulated radiotherapy (IMRT) is the preferred techniques, while combination with chemotherapy is another treatment of locally advanced disease [11]. IMRT revealed excellent results with more than 85% locoregional control and the prognosis of NPC is relatively better than all other head and neck cancers. However, lymph node metastasis or distant metastasis remains the most difficult condition [12]. Hence, more effective systemic agents which can inhibit metastasis of NPC should be sought.

Many steps are critical to metastasis, including angiogenesis, invasion of the blood vessels, invasion of or migration through of the basement membrane (BM) and extracellular matrix (ECM), and adhesion to the target organ site [13]. Matrix metalloproteinases (MMPs) are endopeptidases with the ability to degrade the basement membrane and ECM. MMPs are also responsible for tumor growth, invasion, and metastasis in many human cancers [14,15]. Furthermore, previous study revealed that higher levels of plasma MMP-2 expression was responsible to lymph node metastasis and poor survival and in NPC patients [16].

From previous studies, many natural compounds provide anti-metastasis ability in NPC. For example, epigallocatechin gallate (EGCG) (0–50 μM), a major green tea polyphenol, decreased tumor proliferation and invasion and induced apoptosis in NPC [17]. *Rubus idaeus* extract (RIE) (0–100 μg/mL) has a chemopreventive effect on NPC by suppressing the expression of MMP-2 through the ERK1/2 pathway [18]. Morusin is also a natural chemotherapeutic agent in many human cancers. However, the relationship and detailed mechanism of morusin in NPC is still unclear. Thus, this study aimed to investigate the anti-tumoral properties of the morusin on NPC cells and elucidate the underlying mechanism.

## 2. Results

### 2.1. Effects of Morusin on the Viability of NPC Cells

**Figure 1:**
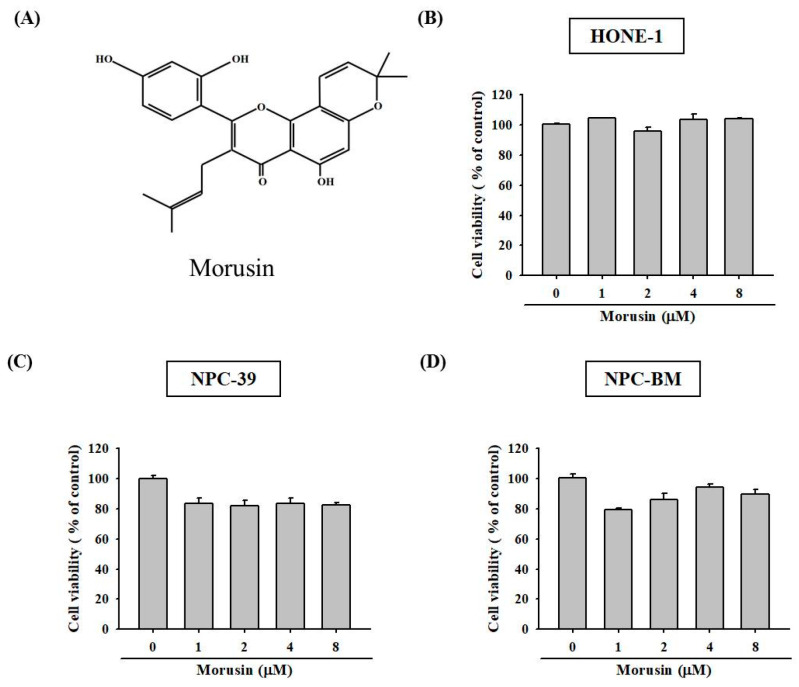
Effect of morusin on cell viability in human NPC cells. (**A**) The chemical structure of morusin. (**B**) HONE-1; (**C**) NPC-39; and (**D**) NPC-BM cells were treated with various concentrations (0–8 μM) of morusin for 24 h and then examined for cell viability. The data are presented as mean ± SD from at least triplicates independent experiments.

To determine whether morusin has cytotoxic effects on NPC cells, HONE-1, NPC-39, and NPC-BM cells were treated with morusin at different concentration (0, 1, 2, 4, and 8 μM) followed by MTT assay. The results show that the viability of HONE-1, NPC-39, and NPC-BM cells was not influenced by the morusin treatment in concentrations ranging from 0 to 8 μM (Figure 1B–D). We applied this concentration range of morusin (0–8 μM) for the following assays.

### 2.2. Effects of Morusin on NPC Cell Migration and Invasion

**Figure 2:**
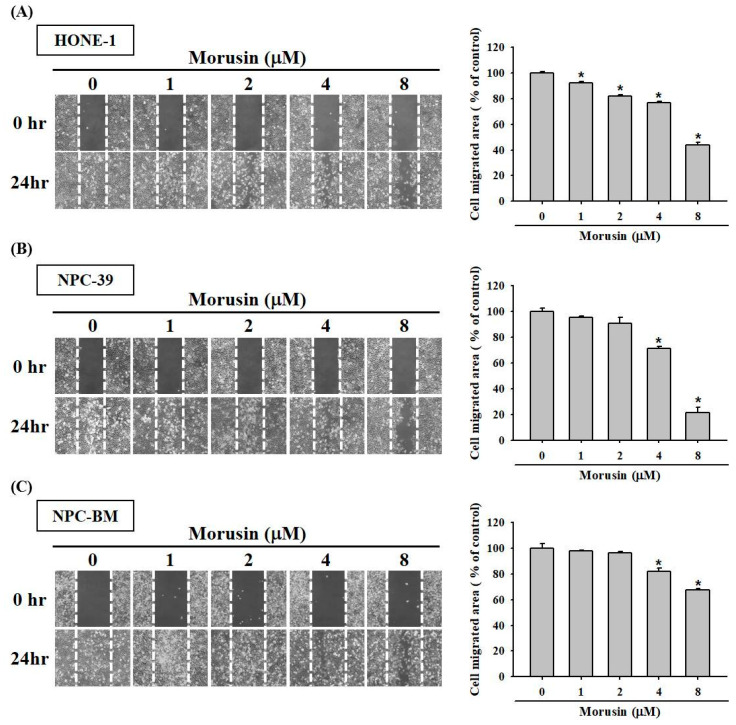
Effect of morusin on cell wound closure in human NPC cells. Wound healing assay evaluated the effect of morusin on cell motility of HONE-1 (**A**), NPC-39 (**B**) and NPC-BM (**C**) cells lines at various concentrations by microscope (at 100× magnification). The data are presented as mean ± SD from at least triplicates independent experiments. * *p* < 0.05 compared with untreated.

**Figure 3:**
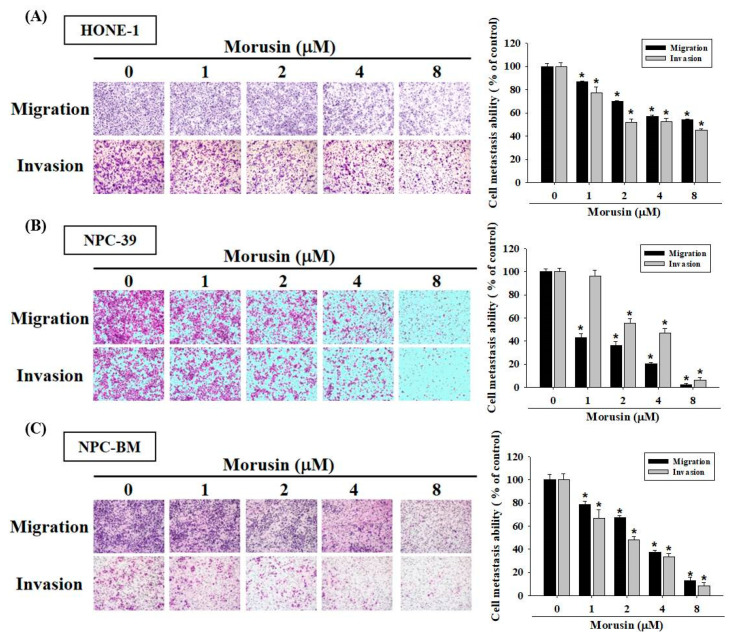
Morusin suppresses the cell migration and invasion of NPC cells. (**A**) HONE-1; (**B**) NPC-39; and (**C**) NPC-BM cells were pretreated with indicated concentrations of morusin. Cell migration and invasion were assayed at 24 h after seeding in a modified Boyden chamber with and without Matrigel coating, respectively. The number of cells was counted using a microscope at 100× magnification. Quantitative data are shown in the right panel. * *p* < 0.05 as compared with morusin-untreated controls.

To examine the effects of morusin on the cell migration and invasion of different NPC cell lines, we used the wound-healing assay and Boyden chamber assay to investigate the cell migration and invasion abilities. In wound-healing assay for HONE-1, NPC-39, and NPC-BM cell lines, the inhibitory effects of morusin on NPC cells migration after 24 h increased as the concentrations of morusin increased (Figure 2A–C). To further evaluate the effects of morusin on the metastatic ability of NPC cells, Boyden chamber assay was performed to measure the effects on the migration (seeding cells with uncoated filter) and invasion (seeding cells with Matrigel-coated filter) activities. As shown in Figure 3A–C, at a morusin concentration of 8 μM, the migration activities for HONE-1, NPC-39, and NPC-BM cells were reduced by 45.9%, 97.8%, and 86.8%, respectively. Moreover, the inhibitory effect of morusin on NPC cell invasion increased as the concentrations of morusin increased. These results demonstrate that treatment with morusin can inhibit the migration and invasion abilities of NPC cells.

### 2.3. Effects of Morusin on MMP-2 Enzyme Activity and Protein Expression

**Figure 4:**
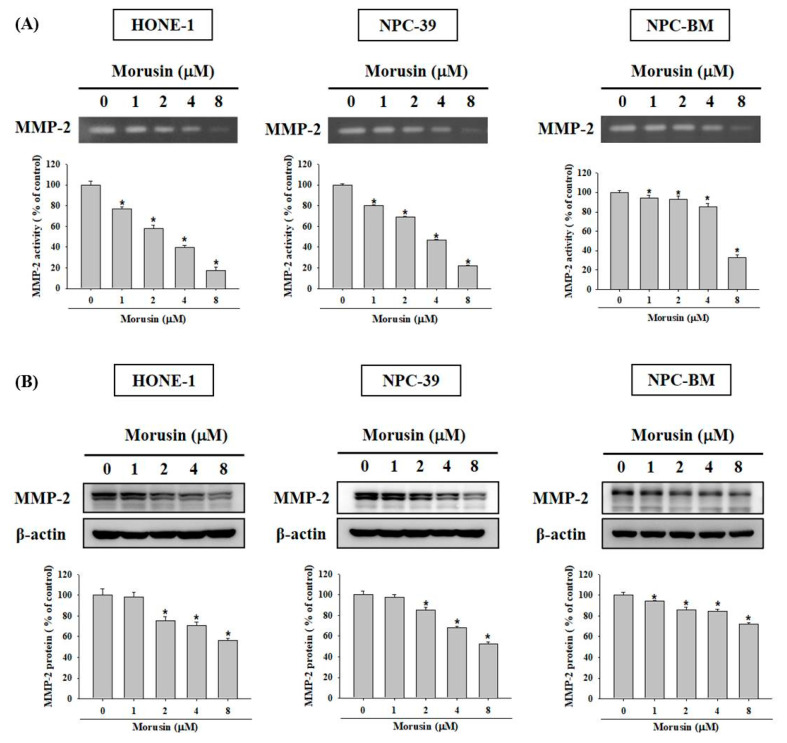
Morusin inhibits the activity and expression of MMP-2 in NPC cells. HONE-1, NPC-39, and NPC-BM cells were treated with morusin (0–8 μM) for 24 h (**A**) Conditioned media were subjected to gelatin zymography for analyzing the activity of MMP-2. (**B**) Total cell lysates were prepared for determining the levels of MMP-2 protein. * *p* < 0.05, compared with the untreated control.

**Figure A1:**
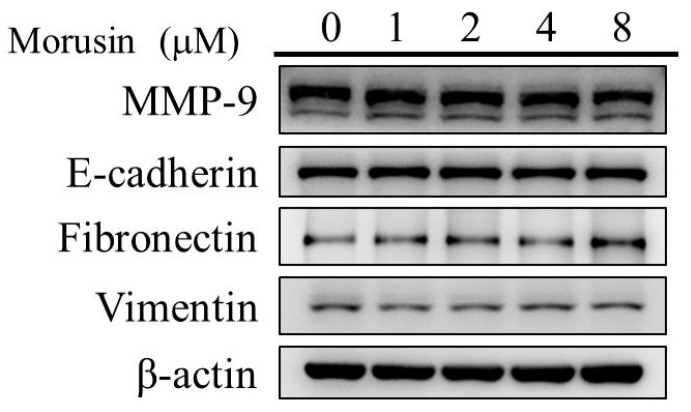
Screening of the inhibitory effects of morusin on multiple protease expressions in NPC-39 cells. Total cell lysates were prepared for determining the levels of MMP-9, E-cadherin, fibronectin, and vimentin protein.

MMP-2, MMP-9 and epithelial-mesenchymal transition (EMT) markers were reported to be correlated with NPC metastasis [19,20,21,22]. We further confirmed expressions of these proteases by performing a Western blotting analysis. After morusin (0, 1, 2, 4, and 8 μM) treatment, the protein expression of MMP-9, E-cadherin, fibronectin and vimentin were not altered in NPC-39 cell lines (Appendix A Figure A1). However, as shown in Figure 4A, the results indicate that morusin efficiently suppressed the MMP-2 activity in the three NPC cell lines. Western blotting assay was performed and demonstrated a reduced expression of MMP-2 protein in the three NPC cell lines when treated with morusin (Figure 4B). These results suggest that morusin could reduce the MMP-2 activity and expression in NPC cell lines.

### 2.4. Effects of Morusin on the MAP-Kinase Signaling Pathways

**Figure 5:**
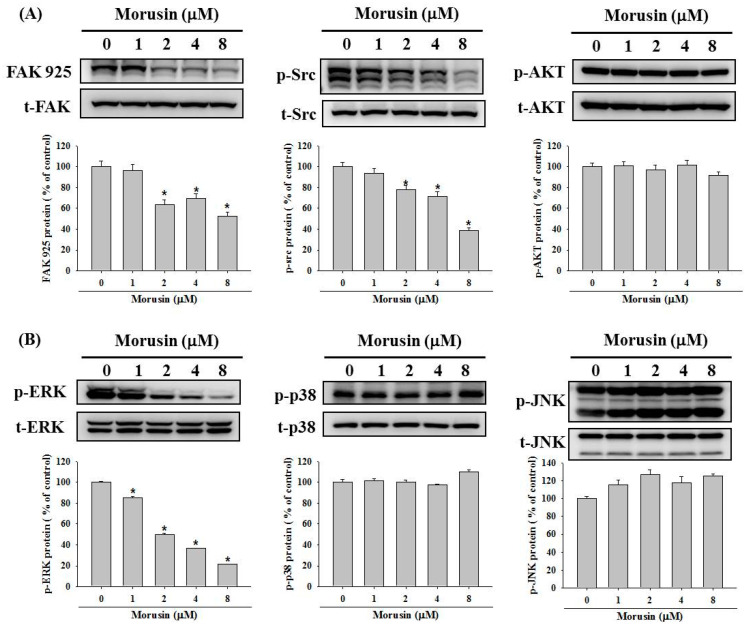
Effect of morusin on regulating the adhesion and MAPK signaling pathways. NPC-39 cells were treated with morusin (0–8 μM) for 24 h and cell lysates were subjected to Western blotting analysis to analyze the phosphorylation of: FAK, Src, and Akt for adhesion signaling (**A**); and ERK, JNK, and p38 for MAPK pathways (**B**). Densitometric analyses of kinase phosphorylation were conducted by ImageJ. * *p* < 0.05, compared with the vehicle group.

**Figure 6:**
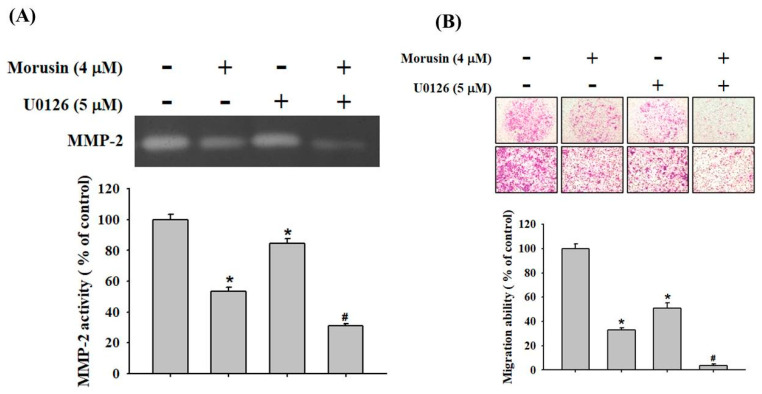
Inhibitory effect of ERK1/2 inhibitor (U0126) on MMP-2 activity and cell migration. (**A**) Human NPC-39 cell lines were pre-treated with U0126 (5 μM) for 1 h and then incubated in the presence or absence of morusin (4 μM) for 24 h, and the conditioned media were subjected to gelatin zymography for analyzing the activity of MMP-2. (**B**) The migration ability of cells was observed by Boyden chamber assay. * *p* < 0.05, compared with the vehicle group. # *p* < 0.05, compared with the U0126 treated group.

Since the Mitogen-activated protein kinases (MAPK) are involved in regulating MMP-2, which is known as a metastasis pathway [23,24], the effects of morusin on the expressions of MAPK pathway were investigated by Western blotting assay. The results demonstrate that morusin significantly inhibited the phosphorylation of FAK, Src, and ERK1/2 in the NPC-39 cell lines, while no effect on the phosphorylation of AKT, p38, and JNK1/2 was measured (Figure 5A,B). To verify whether the inhibition of MMP-2 activity by morusin was caused by the downregulation of phosphorylated ERK1/2, the effects of U0126, an inhibitor of the ERK1/2 signaling pathway, on the MMP-2 activity and cell migration for NPC-39 cells was examined. As shown in Figure 6A, B, the combination treatment of morusin (4 μM) and U0126 (5 μM) resulted in the intense inhibition of the MMP-2 activity and cell migratory ability in NPC-39 cells.

## 3. Discussion

Nasopharyngeal carcinoma is a unique type cancer because of the specific geographical distribution. Most of the new cases occurred in Southern China and Southeast Asia. Locoregional failure or distant metastasis remains the most difficult challenge at present [11]. Knowing the mechanism of NPC metastasis and new pharmaceuticals natural products with the anti-tumoral ability were important. To understand the anti-tumoral effect of morusin on NPC cells, three epithelial NPC cell lines, HONE-1, NPC-39, and NPC-BM, were used. Our results show that morusin can suppress the metastatic ability of these NPC cells effectively.

Prenylated flavonoids are famous in Asia due to their benefits for human health. They are considered important sources of new medicines for their anti-microbial and anti-inflammatory activities [4]. Morusin is one of the prenylated flavonoids which have antitumor activity against many human cancers. In many prior studies, morusin was reportedly to have chemopreventive effects against gastric cancer, prostate cancer, colorectal cancer, and breast cancer by different mechanisms [5,6,7,8]. Up to now, there is no research about the relationship between the morusin and NPC. From the current study, morusin can suppress the migration and invasion of human NPC cells. These results indicate a usefulness of morusin on restraining NPC invasion and metastasis. Morusin has weak cytostatic effects on human cells at a concentration below 8 μM, suggesting that experimental concentrations of the extract should be feasible in the clinical setting and it is recommended for NPC patients to take morusin as a chemopreventive agent. Several previous studies showed the chemopreventive effect of morusin against the human cancer by some possible mechanisms, including induction of apoptosis in cancer cells and inhibition of cell proliferation and tumor growth [5,6,7,8]. On the other hand, our results demonstrate that morusin suppresses metastatic activity of NPC cells by downregulating the protein expression of MMP-2.

There are several hallmarks of cancer, such as avoiding immune destruction, tumor promoting inflammation, resisting cell death, and activating invasion and metastasis [25]. Since regional and distant metastasis are the most important and difficult conditions for the NPC patients, activating invasion and metastasis are the critical steps. Metastasis is composed of many molecular interactions, one of them being the degradation of extracellular matrix and basement membrane of the vessel endothelium [14,15,26]. MMP family plays an important role in degrading components of the ECM and basement membrane [13]. Over 25 MMP members have been identified; MMP-2 and MMP-9 are the most important proteins in metastasis. When a tumor first becomes vascularized (angiogenic switch), MMP-2 and MMP-9 have been shown to be critical for tumor angiogenesis. However, our Western blotting data show that only MMP-2, but not MMP-9, protein expression was inhibited by morusin in a concentration-dependent manner. Downregulation of MMP-2 results in loss of angiogenic potential and inhibits tumor cell migration [27]. MMP-2 is well known not only for its degradation of ECM, but also as a tumor marker for poor prognosis of head and neck cancer [28]. Furthermore, from the previous study, MMP-2 is not only a tumor marker in NPC, but also related to lymph node metastasis and poor survival outcome. This suggests MMP-2 is a notable factor for metastasis in NPC [29,30]. We therefore investigated whether MMP-2 is involved in the anti-tumoral activity of morusin on NPC cells. Our results reveal that morusin inhibits the migration and invasion abilities of NPC cells by suppressing the protein expression of MMP-2. Previous studies have demonstrated that inhibition of MMP activity can be used as an early target to prevent cancer metastasis [28,31]. Thus, morusin may serve as a potential agent for cancer therapy.

To understand the precise molecular mechanism of the metastatic suppression abilities on NPC cells by downregulation of MMP-2 of morusin, our study investigated the signaling pathway regulating the anti-tumoral effects of morusin. Mitogen-activated protein kinases (MAPKs), a family of serine-threonine protein kinases involved in cytoplasmic and nuclear effectors which regulate may cellular functions, are important in cell growth, differentiation, migration, and apoptosis [32,33]. The phosphorylation of major MAPK proteins, including Akt, p38, ERK1/2, and JNK, was measured by Western blotting. Our data reveal that morusin decreased the phosphorylation of ERK1/2 protein while the other proteins were not involved in the process. From previous study, p-ERK1/2 and Ki-67 are highly expressed in nasopharyngeal carcinoma patients and ERK1/2 signaling pathway participates in the NPC proliferation and apoptosis [34]. In another study, pinostilbene hydrate (0–80 μM) inhibited the migration and invasion of NPC cell by downregulation of MMP-2 and epithelial-mesenchymal transition suppression through ERK1/2 signaling pathway [35]. Nobiletin (0–40 μM) inhibited invasion and migration of NPC by regulating the activity of MMP-2 via suppressing NF-kB and AP-1 nuclear translocation and suppressing the phosphorylation of ERK1/2 [36]. Compared with the above studies, our data also show that morusin treatment significantly inhibited the phosphorylation of ERK1/2 which is in controlling the metastatic processes of NPC.

## 4. Materials and Methods

### 4.1. Cells and Cell Culture

HONE-1, one of the human nasopharyngeal carcinoma cell lines, was obtained from ATCC (Manassas, VA, USA). NPC-39 and NPC-BM were given by Dr. MK Chen, Department of Otolaryngology, Changhua Christian Hospital, Changhua, Taiwan. All three cell lines were cultured in RPMI-1640 medium and maintained at 37 °C in a humidified atmosphere of 5% CO_2_.

### 4.2. Microculture Tetrazolium (MTT) Assay

To evaluate the cytotoxicity of morusin (No. CFN97083, Purity ≥ 98%, ChemFaces Biochemical Co., Ltd. Wuhan, China), microculture tetrazolium (MTT) colorimetric assay was performed to determine cell viability, as previously described [37]. After Formazan formed, and the samples were measured spectrophotometrically (Bio-Tek Instruments, Winooski, VT, USA) at 570 nm.

### 4.3. Wound Healing Assay

Human nasopharyngeal carcinoma cell lines, HONE-1, NPC-39, and NPC-BM, were seeded in six-well plates for 24 h with 8 × 10^5^ cells/well and the cells started overnight. The cells were wounded by scratching with a yellow tip, incubated with a condition medium containing 0.5% FBS, and subsequently received various treatments of morusin (dissolved in dimethyl sulfoxide) (0, 1, 2, 4, and 8 μM). Cell migration was photographed at 0 and 24 h by using an Olympus CKX41 phase contrast microscope (Olympus Corporation, Tokyo, Japan) at 100× magnification.

### 4.4. Cell Migration and Invasion Assays

Cell migration and invasion were assayed according to the methods described by Lin et al. [38]. After treatments with morusin (0, 1, 2, 4, and 8 μM) or Erk1/2 inhibitor (U0126) for 24 h, the surviving NPC cells were harvested and seeded to the upper Boyden chamber (Neuro Probe, Cabin John, MD, USA) at 10^4^ cells/well in serum-free medium. After 24 h incubation at 37 °C, the cells were fixed by 100% methanol and stained with 10% Giemsa stain for 3 h. To determine cell migration, the cells were seeded into the Boyden chamber on membrane filters that were not coated with Matrigel, as described by Lin et al. [38]. The number of cells was counted using an Olympus CKX41 microscope (Olympus Corporation, Tokyo, Japan).

### 4.5. Determining MMP-2 Activity through Gelatin Zymography

Gelatin zymography was used to determine the activities of MMP-2 in the conditional medium. After treatments with morusin (0, 1, 2, 4, and 8 μM) in serum-free medium for 24 h, collected medium containing 10 μg of total protein were prepared with SDS sample buffer without boiling or reduction, and subjected to 0.1% gelatin −8% SDS-PAGE electrophoresis. After SDS-PAGE electrophoresis, the gels were washed with 2.5% Triton X-100 and incubated in a reaction buffer (40 mM Tris–HCl, pH 8.0; 10 mM CaCl_2_ and 0.01% NaN_3_) at 37 °C for 12 h. The gel was stained with Coomassie brilliant blue R-250 for visualization.

### 4.6. Western Blotting Analysis

Total cell lysate was analyzed by SDS-PAGE in 10% polyacrylamide gel and transferred onto a nitrocellulose membrane as previously described [39]. Anti-MMP-2, ERK, phospho-ERK, phospho-Src, phospho-Y925-FAK, phospho-p38, JNK, phospho-JNK, and phospho-AKT antibodies were purchased from Cell Signaling Technology (Danvers, MA, USA).

### 4.7. Statistical Analysis

Statistically significant differences were calculated using the one-way ANOVA followed by Tukey’s test. When two groups were compared, the data were analyzed by using Student’s *t*-test. Significance was set at *p* < 0.05. The presented values are the means ± standard deviation (SD) of at least three independent experiments.

## 5. Conclusions

Our study demonstrated that morusin inhibits the invasion and metastatic activity of human NPC cells by suppressing the expression and activity of MMP-2 by downregulating the ERK1/2 signaling pathway. Morusin can exert inhibitory effects in NPC metastasis and may be a potential chemotherapeutic agent for patients with NPC.
